# Trichodysplasia Spinulosa Polyomavirus in Respiratory Tract of Immunocompromised Child

**DOI:** 10.3201/eid2409.180829

**Published:** 2018-09

**Authors:** Arwa A. Bagasi, Tasneem Khandaker, Gemma Clark, Terry Akagha, Jonathan K. Ball, William L. Irving, C. Patrick McClure

**Affiliations:** King Saud University, Riyadh, Saudi Arabia (A.A. Bagasi);; University of Nottingham, Nottingham, UK (A.A. Bagasi, T. Khandaker, T. Akagha, J.K. Ball, W.L. Irving, C.P. McClure);; Nottingham University Hospitals NHS Trust, Nottingham (G. Clark, W.L. Irving)

**Keywords:** TSPyV, trichodysplasia spinulosa, trichodysplasia spinulosa polyomavirus, PCR, respiratory tract, polyomavirus, human polyomavirus 8, viruses, child, United Kingdom, respiratory infections

## Abstract

Trichodysplasia spinulosa polyomavirus causes trichodysplasia spinulosa, a skin infection, in immunocompromised persons, but the virus is rarely detected in respiratory samples. Using PCR, we detected persistent virus in respiratory and skin samples from an immunocompromised boy with respiratory signs but no characteristic skin spicules. This virus may play a role in respiratory illness.

Trichodysplasia spinulosa is a rare skin disease that occurs exclusively in immunocompromised persons. It is characterized by facial keratotic spicules formed by trichohyalin accumulation in the inner root sheath cells of affected hair follicles. In 1999, electron microscopy identified a novel polyomavirus, subsequently named trichodysplasia spinulosa polyomavirus (TSPyV) or human polyomavirus 8, in sections of skin spicules of a solid organ transplant patient (*1*); in 2010, the virus was more completely characterized (*2*). TSPyV is 1 of 5 polyomaviruses associated with human diseases, particularly those that affect immunocompromised persons (*3*). Although worldwide seroprevalence of TSPyV antibodies among the general population is estimated at 70% (*4*) and a respiratory route of infection has been hypothesized (*5*,*6*), as of 2015, only 32 cases of trichodysplasia spinulosa had been reported (*7*), suggesting that other pathology caused by TSPyV may have gone undiagnosed. We describe PCR detection of TSPyV in an immunocompromised boy with respiratory signs and symptoms.

To elucidate potential causes of undiagnosed viral respiratory infection, during January 2015–February 2016, we used a panpolyomavirus degenerate primer PCR to screen archived samples for polyomavirus. The archived samples were nucleic acid of respiratory specimens from 218 children 6 months to 5 years of age, previously negative for typical respiratory viruses in a panel used for routine diagnosis (Technical Appendix). Of the 218 samples screened in 22 pools, we obtained positive results for polyomavirus in 1 pool and, subsequently, 1 sample (from the patient reported here). Subsequent Sanger sequencing and BLAST (http://blast.ncbi.nlm.nih.gov/Blast.cgi) analysis of the 274-bp degenerate primer PCR product indicated that the sample contained TSPyV. The complete genome of this TSPyV strain was amplified in 4 overlapping PCR fragments and Sanger sequenced (Technical Appendix). Phylogenetic analysis of the assembled complete 5,232-nt genome with all available 23 reference sequences revealed that the TSPyV strain was most closely related to TSPyV 1312, which had been isolated in 2012 in Dallas, Texas, USA (Technical Appendix), but bootstrap support was limited because of the highly conserved nature of TSPyV genomes.

The patient from whom this TSPyV-positive sample was collected was a 4-year-old boy in Nottinghamshire, United Kingdom, who had common acute lymphoblastic leukemia and was receiving maintenance chemotherapy during the study period. Retrospective clinical analysis for March 2014–February 2016 revealed that the child had had frequent cough with fever and coryzal symptoms of varying severity (Table). Concurrently collected nasopharyngeal aspirate and throat swab specimens were negative for bacterial and viral pathogens routinely tested for, except at the start of the study period, when rhinovirus and adenovirus were detected, and the end of the period, when rhinovirus and respiratory syncytial virus were detected (Table). No bacteria were cultured from paired specimens. On this basis, in conjunction with unremarkable physical examination and radiologic findings and stable neutrophil and leukocyte counts (data not shown), the patient’s respiratory signs were treated conservatively on an outpatient basis. However, on 2 occasions (August and November 2015), the child required hospital admission, without and with co-infection, respectively. 

**Table:** Clinical and laboratory data from TSPyV-positive patient, Nottinghamshire, United Kingdom, November 2014–2015*

Collection date	Sample type	Signs and symptoms at time of sample collection	Documented skin lesion	Hospital admission	Viral/bacterial co-infection	TSPyV C_t_ value
2014						
Nov	Throat swab	Cough, sore throat, fever	Tiny skin colored pustules on hand	Not required	Rhinovirus	31.83
Dec	NPA	Cough, fever	None	Not required	Adenovirus	31.17
2015						
Jan	Skin swab	None recorded	Suspected varicella zoster virus rash	Not required	None	24.97
Feb	NPA	Dry cough, fever	None	Not required	None	31.23
Mar	Throat swab	Dry cough, coryzal symptoms	None	Not required	None	21.43
Jul†	Throat swab	Cough with runny nose	None	Not required	None	23.90
Jul†	NPA	Cough with runny nose	None	Not required	None	22.70
Jul‡	NPA	Dry cough, fever	Few blisters on fingers	Not required	None	22.30
Jul‡	Throat swab	Dry cough, fever	Few blisters on fingers	Not required	None	25.37
Aug	Throat swab	Cough, fever (high)	Erythematous rash with tiny white center on face	Hospitalized 4 d	None	21.47
Sep	NPA	Cough	None	Not required	Rhinovirus	26.87
Nov	Throat swab	Cough, wheeze, fever (high), coryzal symptoms	Rash across chest	Hospitalized 5 d	Respiratory syncytial virus	23.45

*C_t_ , cycle threshold; NPA, nasopharyngeal aspirate; TSPyV, trichodysplasia spinulosa polyomavirus; VZV, varicella zoster virus. †Collected on the same date. ‡Collected on the same date.

Further retrospective laboratory investigation found that all 11 additional samples collected from this patient during November 2014–2015 were positive for TSPyV, with co-infection at the 4 time points (November and December 2014, September and November 2015); testing showed fluctuating cycle threshold (C_t_) levels on quantitative PCR (Table; Technical Appendix). Of note, various forms of rashes appeared in different anatomic regions of the patient but did not resemble the characteristic appearance of trichodysplasia spinulosa and, thus, did not raise any clinical suspicion for this condition. Indeed, retrospective testing found that a single skin swab sample taken from a suspected viral rash (site undocumented) that looked like blisters and was queried as chickenpox was positive for TSPyV with a low C_t_ value of 24.97 (Table). Thus, it is conceivable that this rash represented the early papular stages of a trichodysplasia spinulosa lesion that did not progress to the characteristic spicules.

Previously, TSPyV has almost exclusively been associated with pathology of the skin (*4*); but 4 reports indicate its isolation from blood (*6*) and respiratory samples, suggesting a potential transmission route (*5*,*8*–*10*). However, respiratory signs and symptoms were observed only in patients co-infected with another virus. In contrast, the patient we report had persistent respiratory signs and symptoms and concomitant TSPyV-positive (by PCR) respiratory samples in conjunction with varying forms of skin lesion lacking the characteristic spicule form of trichodysplasia spinulosa. However, it is difficult to assess the virus pathogenicity in the absence of any supportive cell culture results. Hence, the potential of TSPyV to cause respiratory signs and symptoms needs further investigation and surveillance. The relatively low C_t_ values (and thus high viral loads) of TSPyV DNA obtained from this patient in the absence of positive results for any other microbial agents may suggest an etiologic role of the TSPyV in respiratory pathogenesis. The fact that TSPyV skin disease can be effectively treated with antiviral medication, such as cidofovir (*6*), presents potential for treatment of respiratory manifestations of TSPyV infection.

Technical Appendix Supplementary methods, primers for trichodysplasia spinulosa polyomavirus amplification, and phylogenetic tree of the complete 5,232-bp trichodysplasia spinulosa–associated polyomavirus genome.
