# Supplementary material for: Trichodysplasia Spinulosa Polyomavirus in Respiratory Tract of Immunocompromised Child
Source: Emerg Infect Dis. 2018 Sep;24(9):1744–6. doi: 10.3201/eid2409.180829 (PMC6106430; doi:10.3201/eid2409.180829)
Supplement: Technical Appendix — Supplementary methods, primers for trichodysplasia spinulosa polyomavirus amplification, and phylogenetic tree of the complete 5,232-bp trichodysplasia spinulosa–associated polyomavirus genome. [file 18-0829-Techapp-s1.pdf]

Article DOI: https://doi.org/10.3201/eid2409.180829

Trichodysplasia Spinulosa Polyomavirus in Respiratory Tract of Immunocompromised Child

Technical Appendix

Supplementary Information

Nucleic acids (NA) were extracted from respiratory specimens of symptomatic individuals referred to Nottingham University Hospitals Trust, Nottingham, UK by the Nuclisens EasyMAG system (Biomerieux). Routine diagnostic PCR / RT-PCR assays for Human Adenovirus, Influenza (A and B), Human Metapneumovirus, Parainfluenza (1–4), Rhinovirus and Human Respiratory Syncytial Virus (RSV, A and B) were undertaken and surplus NA archived at −72°C. A total of 198 NPA and 20 throat swabs negative for the aforementioned viruses were identified from patients aged 6 months to 5 years old between January 2015 and February 2016 and subjected to extended diagnostic tests. All subsequently analyzed longitudinal samples from the sole TSPyV positive individual identified had been subject to the same diagnostic pathway, with the exception of January 2015 skin swab. This sample was screened by PCR / RT-PCR for Herpes Simplex Virus 1 and 2, Varicella Zoster Virus, Mumps virus, Enterovirus and Parechovirus and found to be negative for all targets.

Five µl NA from 8 −10 samples were combined to make a total of 22 pools and 1 µl of each screened for Polyomaviruses using a previously described degenerate pan-*Polyomavirus* assay (*1*) modified by using only the VP1–2 primer pair (see table below) and HotStarTaq (QIAGEN) in 15 µl reactions with the following PCR cycle conditions: 95°C for 15 minutes then 55 cycles of 95°C for 20s, 57°C for 20s and 72°C for 20s. Constituents of any positive pools were then re-screened individually to isolate the positive sample component(s). All putative positive samples were confirmed by Sanger sequencing (Source Bioscience, Nottingham UK) and BLAST analysis of the resulting nucleotide sequences (https://blast.ncbi.nlm.nih.gov/Blast.cgi).

TSPyV-positive samples were quantitatively investigated by SYBR green Realtime PCR (qPCR) using novel in-house primers TSPyV326f and TSPyV510r (see table below, PCR conditions as above, Ct values represent an average of triplicate values) designed in Primer3 (http://primer3.ut.ee/) using an alignment created in MEGA7 (http://www.megasoftware.net/) of all 23 complete reference sequences available on Genbank in March 2018 (https://www.ncbi.nlm.nih.gov/nuccore/?term = txid862909[Organism:noexp]) to target a highly conserved genomic region. The entire circular 5232 nt genome of a single TSPyV-positive sample was amplified in 4 overlapping PCR products using additional primers designed as above (see table below, PCR conditions as above except with a 2 minute extension step and 45 cycles only). Amplicons were Sanger sequenced using both forward and reverse primers and a contiguous sequence representing the complete genome (Genbank ID MH351677) was assembled using Seqman Pro (DNASTAR, Madison WI) and compared to the reference genomes in MEGA7 (see figure below).

Reference

1. Johne R, Enderlein D, Nieper H, Müller H. Novel polyomavirus detected in the feces of a chimpanzee by nested broad-spectrum PCR. J Virol. 2005;79:3883–7. PubMed http://dx.doi.org/10.1128/JVI.79.6.3883-3887.2005

Technical Appendix Table. Primers for TSPyV PCR amplification

Assay	Primer	Genome position KU221329	Sequence	Product size	Source
Pan-polyoma screen	VP1–2f<br>VP1–2r	2068–2097<br>2313–2341	ATGAAAATGGGGTTGGCCCNCTNTGYAARG<br>CCCTCATAAACCCGAACYTCYTCHACYTG	274bp	Johne et al 2005 (*1*)
TSPyV specific PCR	TSPyVf326<br>TSPyVr510	326–345<br>491–510	TAACAACACATCCTCCCGCT<br>GCTGCTTCTCCTGCTAACAC	185bp	This study
Full genome amplification	TSPyVf326<br>TSPyVr1902	as above<br>1883–1902	as above<br>GATTGAGGCCTTGTGTGCTC	1577bp	This study
Full genome amplification	TSPyVf1774<br>TSPyVr3176	1774–1793<br>3156–3176	ATATGTTTGCAGTGGGTGGG<br>TGCTCTGATGAATTTCCTGG	1403bp	This study
Full genome amplification	TSPyVf3045<br>TSPyVr4448	3045–3065<br>4428–4448	AGTGCTATTTGACCCTTGACA<br>GGGGTAAGTAAMAGGTGGGGA	1404bp	This study
Full genome amplification	TSPyVf4255<br>TSPyVr510	4255–4274<br>as above	AGAGRTCTGGGTCCTGCATG<br>as above	1488bp	This study

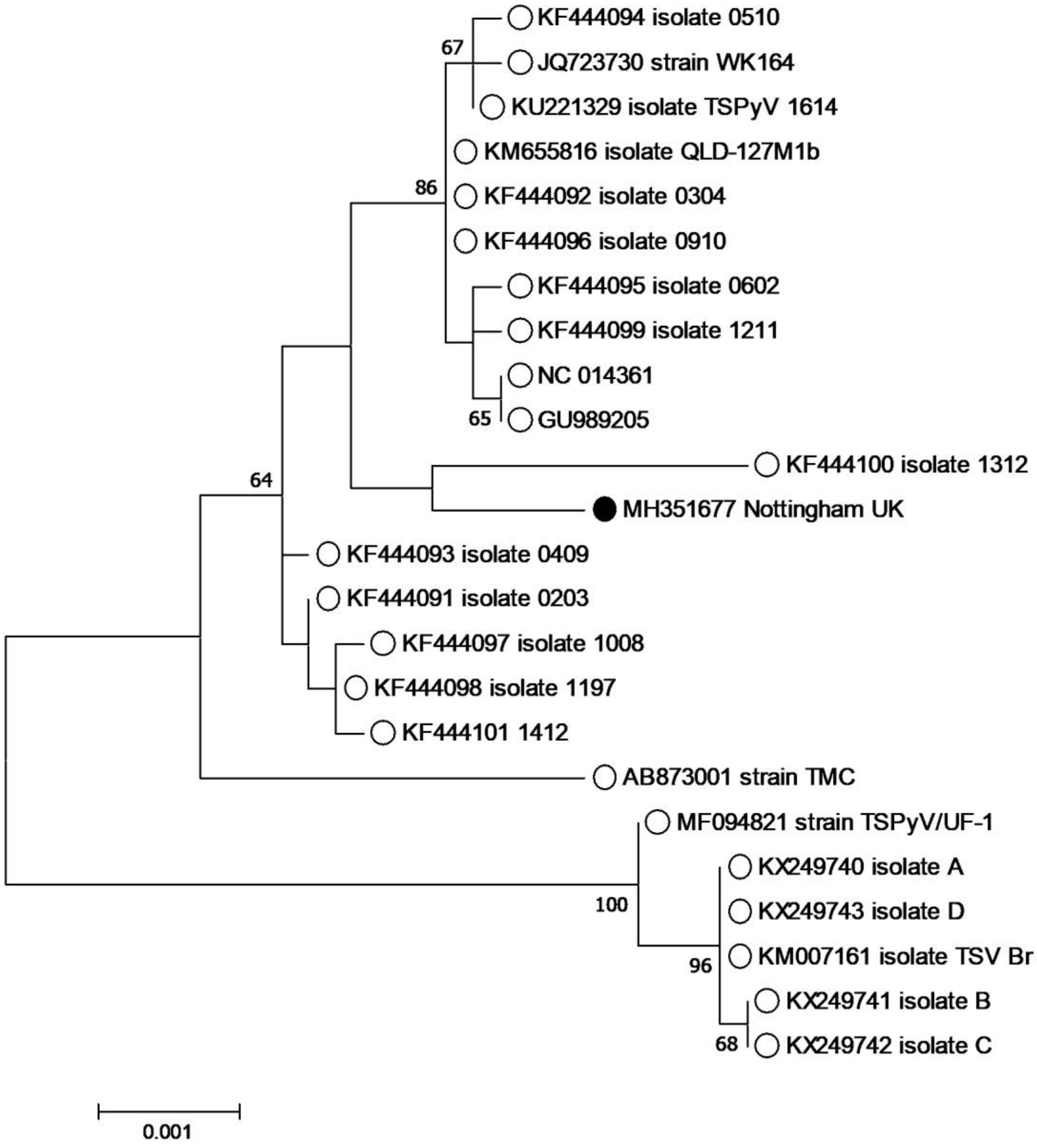

Technical Appendix Figure. Phylogenetic tree of the complete 5232 bp Trichodysplasia Spinulosa-associated Polyomavirus genome. The tree was constructed using the maximum likelihood method based on Tamura-Nei model and 1000 bootstrapping replications. Twenty-three available complete TSPyV genomes (open circles) were retrieved from Genbank in March 2018 and compared with the strain identified in this study (filled circle).
